# Optimization of ZnO Nanorod-Based Surface Enhanced Raman Scattering Substrates for Bio-Applications

**DOI:** 10.3390/nano9030447

**Published:** 2019-03-17

**Authors:** Miyeon Jue, Sanghwa Lee, Bjorn Paulson, Jung-Man Namgoong, Hwan Yeul Yu, Gwanho Kim, Sangmin Jeon, Dong-Myung Shin, Myung-Soo Choo, Jinmyoung Joo, Youngjin Moon, Chan-Gi Pack, Jun Ki Kim

**Affiliations:** 1 Biomedical Engineering Research Center, Asan Medical Center, Seoul 05505, Korea; imascai@naver.com (M.J.); pause1919@gmail.com (S.L.); bjorn.paulson@gmail.com (B.P.); youngjin.moon@gmail.com (Y.M.); 2 Department of Surgery, Asan Medical Center, University of Ulsan College of Medicine, Seoul 05505, Korea; namgoong2940@gmail.com; 3 Department of Urology, Asan Medical Center, University of Ulsan College of Medicine, Seoul 05505, Korea; hwanyel@naver.com (H.Y.Y.); mschoo@amc.seoul.kr (M.-S.C.); 4 Department of Biomedical Sciences, Asan Medical Center, University of Ulsan College of Medicine, Seoul 05505, Korea; d0shin03@amc.seoul.kr; 5 Department of Chemical Engineering, Pohang University of Science and Technology, Pohang 37673, Korea; kgoanho929@postech.ac.kr (G.K.); jeons@postech.ac.kr (S.J.); 6 Department of Biomedical Engineering, Ulsan National Institute of Science and Technology, Ulsan 44919, Korea; jjoo@unist.ac.kr; 7 Department of Convergence Medicine, University of Ulsan College of Medicine, Seoul 05505, Korea; changipack@amc.seoul.kr

**Keywords:** gold coated thickness, ZnO nanorods, surface enhancement Raman spectroscopy (SERS), Au coated SERS, bladder disease detection

## Abstract

Nanorods based on ZnO for surface enhanced Raman spectroscopy are promising for the non-invasive and rapid detection of biomarkers and diagnosis of disease. However, optimization of nanorod and coating parameters is essential to their practical application. With the goal of establishing a baseline for early detection in biological applications, gold-coated ZnO nanorods were grown and coated to form porous structures. Prior to gold deposition, the grown nanorods were 30–50 nm in diameter and 500–600 nm in length. Gold coatings were grown on the nanorod structure to a series of thicknesses between 100 and 300 nm. A gold coating of 200 nm was found to optimize the Rhodamine B model analyte signal, while performance for rat urine depended on the biomarkers to be detected. These results establish design guidelines for future use of Au-ZnO nanorods in the study and early diagnosis of inflammatory diseases.

## 1. Introduction

Raman spectroscopy is a non-destructive, non-invasive measurement method, in which spectra are collected from the inelastic scattering that occurs when light of a specific wavelength is incident on a material. It is useful for quickly validating the components of a biological sample. However, the signal sensitivity of Raman scattering is poor because the probability of inelastic scattering is generally below 1/10^6^ [1]. To overcome this disadvantage, substrate-based surface-enhanced Raman scattering (SERS) methods have been developed. The enhancement factor (EF) of the SERS effect is mainly affected by electromagnetic fields (EM). The EM enhancement is influenced by the local surface plasmon resonance (LSPR), which is in turn caused by the interaction between incident light and the surface electron of noble metal nanoparticles (NPs). When LSPR occurs in a nanoparticle group, a “hot-spot” is generated in the nanogap between nanoparticles, and as a result, EM is greatly improved and the SERS effect is boosted. The enhanced SERS signal is amplified by 10^4^~10^8^ compared to a typical Raman scattering signal [2,3,4,5,6]. For effective SERS, it is important to use a SERS substrate with uniformly arranged metal head nanostructures to optimize the spacing between the metal structures and to generate uniform particles such that the enhancement of LSPR is maximized [7,8,9,10,11].

The metals most commonly used for SERS are gold, silver, and copper [12,13]. However, for biotechnology applications, toxicity testing to determine biocompatibility is essential. As gold has passed cytotoxicity testing in several studies, it can potentially be used in biological applications [11,14]. Various research groups have attempted to improve SERS by controlling the size and shape of gold nanoparticles and have reported good results. However, when gold nanoparticles or colloidal particles are used, it is difficult to control the distance between the nanoparticles, and the uniformity and reproducibility of the entire substrate are low, resulting in irregular results in Raman analysis.

We fabricated zinc oxide (ZnO) nanorods which distribute gold particles uniformly at the same height to increase the reproducibility of the SERS substrate and to obtain consistent analysis results. ZnO is frequently used because it grows economically and at low temperature and because it allows the production of nanostructures of various shapes and sizes [15,16,17]. Moreover, the SERS effect has been reported to become stronger when the ZnO nanomaterial and the plasmon-generating metallic nanoparticles are combined because of the charge transfer interaction between semiconductors and plasmonic metals [18,19,20].

**Figure 1:**
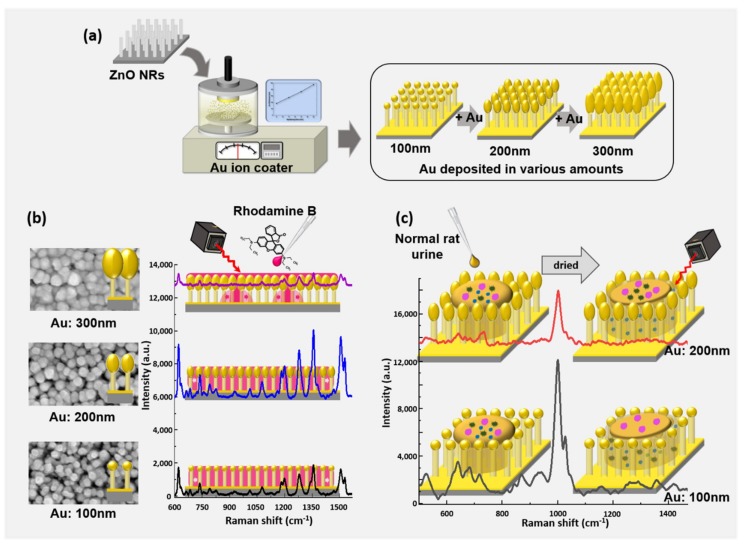
Schematic showing the processes of the ZnO nanorod-based surface-enhanced Raman scattering (SERS) substrate. (**a**) Au coated substrate production, (**b**) optimization of application, and (**c**) measurement of rat urine.

As shown in Figure 1, in this study, healthy rat urine was measured by Raman spectroscopy using SERS substrates optimized for Rhodamine B (Rh B) by depositing measured amounts of Au on ZnO nanorods. Chemical signatures characteristic of biomarkers common to urine were detected from 638 to 1356 cm^−1^, and significant changes in peak strength were observed for individual peaks between the optimized and un-optimized SERS substrates. It was observed that the SERS substrates optimized for detection of Rh B are suboptimal for many biomarkers in urine, although there are a few peaks from urine which were observed with higher intensity using the substrate optimized for Rh B. A physical mechanism is proposed involving the morphology of Au-deposited ZnO nanorods to explain these observations. By demonstrating the detection of biomarkers and determining the pitfalls of signal to noise ratio optimization between biomarkers in biologically important samples, this study lays the groundwork for future optimization of Au-deposited ZnO nanorod SERS substrates to specific and biologically meaningful chemical targets.

## 2. Experimental Methods

### 2.1. SERS Substrate Manufacture

#### 2.1.1. ZnO Nanorod Growth

A porous ZnO nanorod substrate was fabricated in the following manner to obtain an amplified Raman signal: (1) Si wafers were diced to an appropriate size (1 × 1 or 2 × 2 cm^2^). (2) The diced wafers were pre-washed with ethanol and deionized (D.I.) water for 5 min. (3) ZnO was deposited on the pre-washed wafer by radio frequency (RF) sputtering at a power of 100 W for 30 min to form a 30-nm ZnO seed layer. The deposited seed layer was grown into a ZnO nanorod by hydrothermal synthesis. (4) A solution was prepared for ZnO growth by mixing 10 mL of zinc nitrate hexahydrate [Zn(NO_3_)_2_·6H_2_O] (Sigma Aldrich Co., St. Louis, MO, USA) and 0.9 mL of ammonium hydroxide (Sigma Aldrich Co., St. Louis, MO, USA) together with 30 mL of D.I. water. (5) The solution was set at 90 °C and substrates were placed in the aqueous solution for 50 min to grow ZnO nanorods. (6) After growing the ZnO nanorods, substrates were washed well with D.I. water and dried with nitrogen gas. As a final step, Au was deposited on the grown ZnO nanorod substrate in an amount equivalent to 100 nm of thin film thickness [11].

#### 2.1.2. Au Thickness Control

An ion coater (Hoyeon Tech Co., Ltd., Seongnam, Korea) was used to control the amount of gold deposited on SERS substrates that had already been subject to the equivalent of 100 nm gold deposition by thermal evaporation. The amount of gold deposition was controlled by quantifying the deposition current and time and by measuring the cross section after deposition on a bare Si wafer. The deposition conditions were 0.1 mbar of bare pressure and 25 mA deposition current, for deposition at a rate of 50 nm per 11 min. The structural and morphological properties of the samples with gold deposited in various amounts were observed using a field emission scanning electron microscope (FE-SEM) (S-4700, HITACHI, Tokyo, Japan) at 10 kV beam voltage.

### 2.2. SERS Measurement

#### 2.2.1. Analytic Substances

Rhodamine B was used to evaluate the characteristics of the SERS substrate dependent upon the thickness of the Au deposited on the ZnO nanorod. Rh B (Junsei Chemical Co., Ltd., Tokyo, Japan) in powder form was diluted into a 1 mM concentration solution, and one drop (5 µL) of this solution was dropped onto SERS substrate and air-dried. Using a metabolic cage, we also collected urine in a 50-mL tube from a healthy 10-week-old female Sprague–Dawley rat, which was used to further identify potential bio applications.

#### 2.2.2. Raman Measurement Method and Analysis

Raman measurements (FEX-INV, NOST, Seongnam, Korea) were performed using a 0.6 NA, 40× objective lens with a focal spot diameter of approximately 2.4 μm as measured on a CCD. In addition, a 785-nm diode laser was used. The spectra of Rh B samples were measured from 7 points at room temperature and at a spectral resolution of 1 cm^−1^, covering the range of 600–1570 cm^−1^, with an integration time of 50 s. The spectra of healthy rat urine samples were measured from 15 points at room temperature and at a spectral resolution of 1 cm^−1^, covering the range of 465–1475 cm^−1^, with an integration time of 50 s. Using Neutral Density (ND) filters, Rh B and healthy rat urine were irradiated with 0.1 mW and 1 mW of excitation light, respectively. After obtaining Raman measurements, all spectra were post-processed by applying third-order polynomial fitting to remove the background, and by applying the Savitzky–Golay smoothing method using the RAON-Spec program provided by NOST. Spectral image visualizations were processed using the Origin program (Origin 2018b, OriginLab Corp., Northampton, MA, USA).

## 3. Results and Discussion

### 3.1. Morphology of SERS Substrate

**Figure 2:**
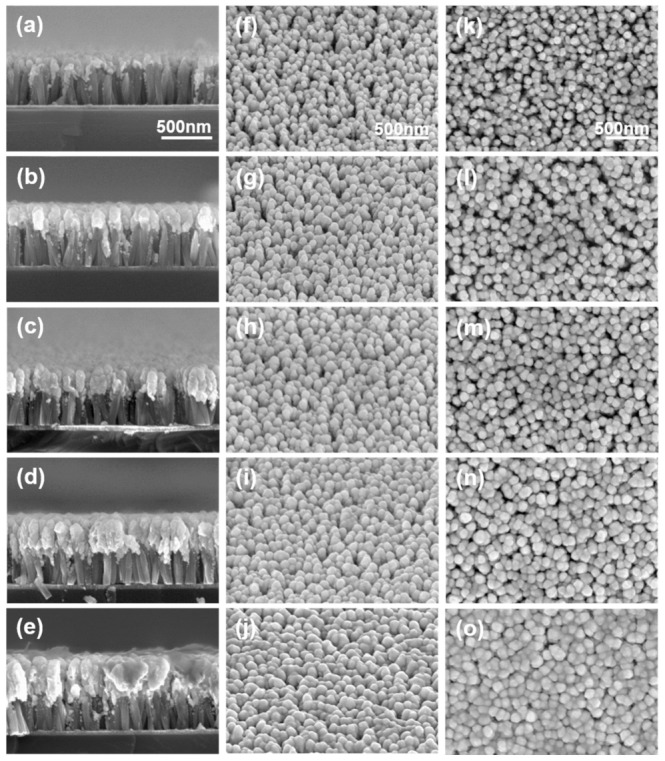
Secondary electron images of the ZnO nanorod substrates show morphology changes based on deposition of different equivalent thickness of gold: (**a**,**f**,**k**) Au: 100 nm; (**b**,**g**,**l**) Au: 150 nm; (**c**,**h**,**m**) Au: 200 nm; (**d**,**i**,**n**) Au: 250 nm; and (**e**,**j**,**o**) Au: 300 nm. (**a**–**e**) cross-sectional images show slight increases in thickness with increased gold deposition. (**f**–**j**) 45-degree tilted images, and (**k**–**o**) plane images show increased filling at higher Au deposition.

To determine the optimal SERS substrate, gold was deposited on ZnO nanorods at thicknesses of 100–300 nm in 50-nm intervals. Figure 2 shows, from left to right, a cross-sectional view, a 45° tilt view, and a plane view, obtained via secondary electron imaging of five SERS substrates. As shown in Figure 2a–e, ZnO nanorods had uniform heights and thicknesses in the range of 500–600 nm and 50–60 nm, respectively, and the deposited gold was concentrated at the head of the ZnO nanorods. In all samples, about 25 nanorods were observed to be randomly distributed on a 500 × 500 nm surface, and the head size and head height of the nanostructures increased with the thickness equivalent of deposited gold. For structures with 100, 150, 200, 250, and 300 nm of gold deposited, the head diameters as viewed from above averaged 74, 90, 100, 120, and 140 nm, and the head heights averaged 99, 181, 237, 294, and 366 nm, respectively (N = 60 each). As the SERS effect depends on the distance between nanostructures, the SERS effect will depend upon the head size when the surface is measured using confocal Raman spectroscopy. As the gold heads on the nanorods increased in size with increased deposition, the space between the heads of adjacent ZnO nanorods became narrower. However, the surface morphology did not change considerably, as shown in Figure 2f–j.

### 3.2. Raman Measurement of Rhodamine B

**Figure 3:**
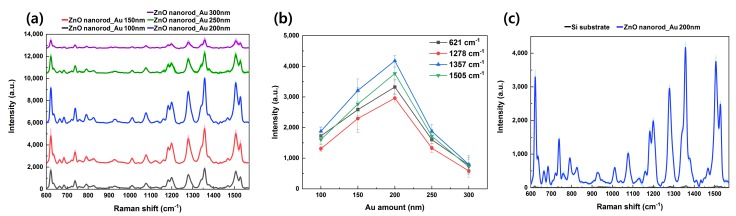
(**a**) SERS spectra and standard deviation (pastel-colored area) of 1 mM Rh B on Au-deposited ZnO nanorods with gold thicknesses of 100 (black curve), 150 (red curve), 200 (blue curve), 250 (green curve), and 300 nm (purple curve). (**b**) Intensity of SERS peaks as a function of the thickness of Au. (**c**) Comparison spectrum of the SERS effect with equivalent illumination power for Rh B on Si substrate (black curve) and on ZnO nanorod substrate deposited with 200 nm gold (blue curve), with standard deviation shown (pastel).

Rhodamine B was selected as a model analyte to investigate the SERS effect of Au-deposited ZnO nanorod substrates. Rh B is a molecule frequently used as a dye in biotechnology, has no optical absorption in the near-infrared region, and is widely used in SERS due to its good adsorption ability with gold nanoparticles [21,22,23]. Figure 3a,b depict the SERS spectra and SERS intensity from Rh B on ZnO nanorod substrates with 100, 150, 200, 250, and 300 nm thicknesses of gold deposition. Figure 3a graphs the SERS mean spectral curves (solid lines) and standard deviations (pastel color) for each Au thickness. The low standard deviation shown in the graph demonstrates the uniformity and reproducibility of the SERS substrates, as well as the absence of measurement variation due to differences in analyte concentration or coffee ring effects; the smaller the standard deviation range, the higher the measurement reproducibility and uniformity.

A SERS intensity graph of the Rh B peaks as a function of gold deposition is shown in Figure 3b. Four peaks in total were selected and plotted. The four peaks are at 621, 1278, 1357, and 1505 cm^−1^, which correspond to the C–C–ring in-plane bend, C–O–C stretching, aromatic ring vibrations, and C–C stretching, respectively [24,25,26]. The SERS intensity increases until the thickness of the gold deposit is 200 nm, and then begins to decrease from when it exceeds 200 nm. This may be attributed to the thickness dependence of the LSPR effect. As the gold-head size gradually increases until the gold deposit amount reaches 200 nm, the head-to-head gap between the gold particles narrows and the LSPR effect increases resulting in a higher SERS intensity; however, if the deposition thickness exceeds 200 nm, local merging of gold heads occurs, which reduces the effect of LSPR and thus decreases the SERS intensity. Figure 3c compares measurements of Rh B on the Si substrate and on the optimized SERS substrate using ZnO nanorod substrates deposited with 200 nm of gold. Measured with identical illumination power, the Rh B measured on the Si substrate showed almost no signal, while the spectra of the optimized SERS substrates show strong signal enhancement and uniform signals. Spectra-to-spectra variations between the 7 measurement points were 13%, 9%, 10% and 13% for the 621, 1278, 1357 and 1505 cm^−1^ peaks, respectively.

### 3.3. Raman Measurement of Healthy Rat Urine

**Figure 4:**
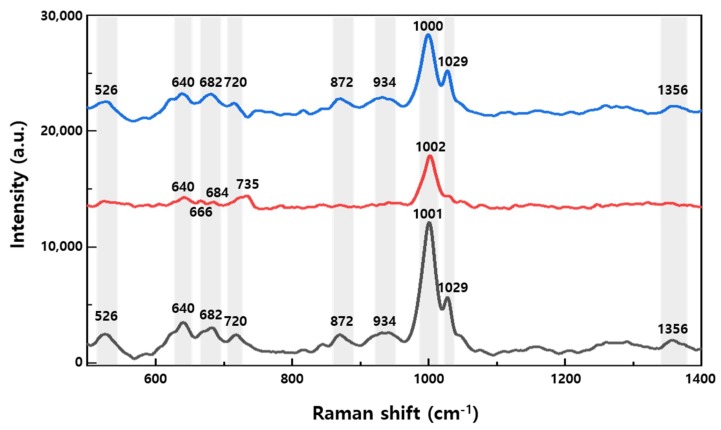
SERS spectra of healthy rat urine measured using 100 nm Au (black curve) and 200 nm Au (red curve) substrates. The blue curve shows the corresponding difference in spectra between the 100 nm gold SERS substrate and the 200 nm gold SERS substrate.

To confirm the bio-applicability of the SERS substrate, rat urine was measured via Raman spectroscopy on ZnO nanorod substrates deposited with amounts of gold equivalent to a thin film thickness of 100 and 200 nm. In life sciences, studies are also underway to diagnose various diseases using the SERS method in urine, blood, and tissues [27,28,29,30]. In Figure 4, the SERS of urine was measured in the range from 465–1475 cm^−1^. The rat urine used was unfiltered urine that contained approximately 25 metabolites. Its Raman spectrum was measured on the SERS substrates using samples that had been fully diffused and dried, and several peaks were observed, as shown in Figure 4. The black graph is the Raman signal of urine from a healthy rat measured on a 100 nm SERS substrate. The peaks for which significant signal was detected were at 526 (corresponding to C–N stretching) [29,31], 638 (C–C twisting mode of tyrosine) [27,28], 682 (ring breathing of nucleic acids for G) [27,32], 720 (ring breathing of nucleic acids for A) [32,33], 872 (C–C stretch of hydroxyproline) [27,28,32,33,34], 934 (C–C stretching mode) [34], 1001 (symmetric ring breathing mode, C–N stretching) [27,28,29,31,32,33,34], 1029 (C–H in-plane bending mode of phenylalanine) [28,34], and 1356 cm^−1^ (CH_3_CH_2_ wagging mode of collagen) [28,34]. The red graph depicts the same sample measured on the 200 nm SERS substrate, and shows a decrease in intensity over the black graph, but additional peaks were resolved at 666 (C–S stretching mode of nucleic acids for T) [27] and 735 cm^−1^ (C–S stretch) [35]. The cause of the signal reduction on the 200 nm SERS substrate is proposed to be the change in the nano-scale gap between Au nanostructures. The increase in the amount of gold deposited increased the diameter of the gold heads, which reduced the size of the nanogap. While a smaller gap is typically associated with higher LSPR enhancement, we suggest that the decreasing size of the nanogaps resulted in larger molecules being blocked from or filtered out by the nanogaps, and thus select SERS signals were reduced. The blue graph shows the difference between averaged spectra on the 100 and 200 nm Au SERS substrates, and thereby the signal due to biomarkers which were filtered out of the 200 nm Au measurement. This result indicates that the optimal conditions of the substrate vary depending on the materials to be measured or the biomarker peaks to be observed. Nonetheless, SERS substrates with Au deposited on ZnO nanorods demonstrate their potential for biological applications.

## 4. Conclusions

Gold-coated ZnO nanorods show promise for the early optical detection and diagnosis of biomarkers via SERS due to their low cost, LSPR-based signal enhancement, and biocompatibility. Raman spectroscopy experiments were conducted with Rhodamine B as a model analyte, which show optimization at a 200 nm thickness of Au coating due to increased LSPR without local fill. Intensity of high sensitivity peaks showed standard deviations between four and five percent, indicating high substrate uniformity. Surprisingly, the SERS substrates optimized for Rh B showed reduced relative detection for most biomarkers in rat urine, although this reduced detection did not hold for all chemical signatures. These results suggest that future Au/ZnO nanorod SERS substrates will benefit from optimization to a subset of individual biomarkers. Nanoscale biomarkers are well dispersed in samples of urine and blood, and despite the benefits of more targeted optimization, the substrates demonstrate the high potential of ZnO nanorods based on SERS for early disease diagnosis.
